# The Role of Social Deficits in the Link Between Social Gambling Motives and Problem Gambling

**DOI:** 10.1007/s10899-025-10374-2

**Published:** 2025-01-18

**Authors:** Christopher G. Floyd, Alexander J. Connolly, Regina K. Tahk, Lindsay M. Stall, Shane W. Kraus, Joshua B. Grubbs

**Affiliations:** 1 Department of Psychology, Bowling Green State University, 822 E. Merry Ave, Bowling Green, OH 43403 USA; 2 University of New Mexico, 2650 Yale BLVD SE, Albuquerque, NM USA; 3 Department of Psychology, University of South Florida, 4202 E. Folwer Avenue, PCD 4118G, Tampa, FL 33620 USA; 4 Department of Psychology, University of Nevada Las Vegas, CEB 320, 4505 S. Maryland Parkway, Las Vegas, NV 89154 USA; 5 Center on Alcohol, Substance Use, And Addictions (CASAA), University of New Mexico, 2650 Yale BLVD SE, Albuquerque, NM USA

**Keywords:** Social gambling motives, Problem gambling, Loneliness, Relatedness frustration

## Abstract

In comparison to other motives for gambling, social motives (e.g., gambling for social interaction) are often suggested to be the least problematic and, in some cases, even a protective factor for problem gambling. This view is consistent with historical distinctions between ‘social’ versus ‘problem’ gambling. As a result, little research has attempted to identify the circumstances in which social motives are more likely to be associated with risk of problems. Consistent with self-determination theory, the present study examined whether the relationship between social motives and problem gambling varies based on social deficits, such as loneliness and a lack of social connectedness, in a weighted, census matched sample of adults in the U.S. (*N* = 2,835). Findings demonstrate support for the moderating influence of social deficits (i.e., loneliness and relatedness frustration) on the relationship between social gambling motives and problem gambling and provide unique insights into the circumstances in which social motives are more likely associated with gambling problems.

## The Role of Social Deficits in the Link between Social Gambling Motives and Problem Gambling

Over the last decade, the U.S. has seen an unprecedented rise in the accessibility of gambling as a product of the advent of online and mobile betting, as well as recent legislative changes, particularly around sports gambling (Lawn et al., 2020; Winters & Derevensky, 2019). As a result of these ecological factors, researchers have demonstrated a rising interest in various underlying factors thought to contribute to problematic gambling (Allami et al., 2021; Welte et al., 2017). Notably, a subset of gambling research has been dedicated to understanding the motivations for or the reasons why people gamble and the role of gambling motives in the development of problem gambling (Dechant, 2014; Stewart & Zack, 2008; Stewart et al., 2008; Tabri et al., 2022). While there are subtle variations in terms of motive categories outlined within motivational models of gambling across studies, contemporary research generally upholds the following established gambling motives: (1) Social Motives (i.e., socialization or connection with others), (2) Enhancement Motives (i.e., induce positive emotions, such excitement or thrill), (3) Coping Motives (i.e., to escape or alleviate negative emotions), and more recently, (4) Financial Motives (i.e., to win money; Stewart & Zach, 2008; Dechant, 2014; Dechant & Ellery, 2011). Yet, there are often conflicting findings in research literature regarding the risks conferred by each of these motives. The present work seeks to address these controversies by exploring the context in which one type of motive—social motives—may be associated with problem gambling.

## Gambling Motives

Early efforts to identify motives behind gambling behavior drew inspiration from research on motives for alcohol use and abuse (Cooper et al., 1992; Cooper, 1994), as well as literature exploring the influence of positive and negative reinforcement in addiction (Baker et al., 1986; Koob, 2013). As a result, scholars contend that both negative reinforcement (e.g., coping) and positive reinforcement (e.g., enhancement, social interaction, and financial gain) can motivate gambling behavior (Dechant, 2014). Over time, research has demonstrated coping, enhancement, and financial motives as robust predictors of problem gambling (Barrada et al., 2019; Marchica et al., 2020; Tabri et al., 2022), with findings regarding the relationship between social motives and problem gambling being less conclusive.

### Social Motives for Gambling

Social motives for gambling can refer to engaging in gambling to socialize, bond with a group, or form new social connections. This includes participating in gambling activities to connect with others or to enhance existing social relationships. In comparison to other motives for gambling, social motives are thought to be the least problematic (Barrault et al., 2019; Francis et al., 2015; Hagfors et al., 2023; Khazaal et al., 2017; Lambe et al., 2015), and are sometimes even considered a protective factor for problem gambling (Mackinnon et al., 2017). For example, Barrada et al. (2019) found that social motives had a strong negative association with problem gambling severity, as well as with gambling frequency across gambling types. Such findings parallel historical distinctions between ‘*social*’ versus ‘*problem*’ gamblers (Huang et al., 2007), in which those who gamble for social reasons are less likely to experience problems (Stewart & Zack, 2008). Such is also consistent with the *Pathways Model* of problem gambling, which suggests that those who gamble for social reasons (i.e., pathway 1’behaviorally conditioned’ cluster), show the lowest problem gambling severity scores (Nower et al., 2022). Still, some studies do indicate that social motives are higher among at-risk and problem gamblers in comparison to non-problem gamblers (Grande Gosende et al., 2019; Jauregui et al., 2020; Savolainen et al., 2022) and demonstrate social motives to be a predictor of problem gambling severity (Estévez et al., 2021; Gökce Yüce et al., 2022; Kim et al., 2019; Shinaprayoon et al., 2017; Mond et al., 2019). Even so, when tested alongside other motives, social motives tend to be a weaker predictor of problem gambling in comparison (Devos et al., 2017; Hagfors et al., 2023 Schellenberg, 2016). Irrespective of whether social motives directly predict problem gambling, what remains unknown is whether the association between social motives and problem gambling might be explained by other variables influencing the relationship. That is, whether the relationship between social motives and risk of problems is contingent on related deficits in basic needs or risk factors, such as a lack of social connectedness and loneliness, which may interact with social motives to increase risk of problem gambling (Hagfors et al., 2023).

## Self-Determination Theory and Basic Psychological Needs

Gambling researchers have pointed to Basic Psychological Needs Theory (BPNT; Ryan & Deci, 2000, 2017), a sub-theory embedded with Self-Determination Theory (SDT), to explain motivating factors that increase vulnerability to gambling problems (Hagfors et al., 2023). SDT suggests the existence of three basic psychological needs, (a) autonomy (i.e., need for self-regulation and control), (b) relatedness (i.e., need for social connectedness), and (c) competence (i.e., need to feel effective and capable), all of which are thought to drive many human behaviors. BPNT posits that when these needs are not properly fulfilled (i.e., need frustration), it can lead to problematic behavioral patterns, such as problem gambling, to satisfy basic needs (Vuorinen et al., 2022). In a recent study, Hagfors et al. (2023) assessed whether frustration of basic psychological needs, as measured by the Basic Psychological Need Satisfaction and Frustration Scale (Chen et al., 2015), moderated the effect of differing gambling motives on problem gambling severity. Results showed that financial motives and need frustration had an interaction effect in which higher need frustration coupled with financial motives predicted greater problem gambling severity. However, in this study, the authors combined all the frustration subscales of the BPNSFS (i.e., autonomy, relatedness, and competence) as opposed to examining the influence of the need categories separately. As such, there is a clear need for gambling studies separately examining satisfaction and frustration of each of the basic psychological needs in the etiology of gambling problems with respect to the contextual factors thought to underlie problem gambling (Vuorinen et al., 2022). One such contextual factor, social deficits, refers to unmet needs for social connectedness, which can manifest in feelings of isolation, loneliness, or a lack of meaningful social support. Social deficits can be operationalized through self-report measures of loneliness and relatedness frustration to examine how social motives may interact with these deficits to increase the risk of gambling problems.

## The Role of Social Context and Loneliness in Gambling

As SDT posits that frustration of the basic psychological needs can lead to maladaptive compensatory behavior, like gambling, there is reason to suspect that social motives would play a greater role in problem gambling behavior when social context does not support the fulfillment of social connectedness. Such is consistent with past works indicating that social deficits, particularly the lack of or need for social connectedness, can lead to excessive gambling as a means of increasing social connection (Nuske et al., 2016). The role of social needs in motivating gambling behavior is also evident in studies indicating that risk of problem gambling increases in the context of loneliness and social isolation (Botterill et al., 2016; Elton-Marshall et al., 2018). While research has consistently demonstrated loneliness as a robust predictor of problem gambling (Hum & Carr, 2018; Khazaal et al., 2017), the link between loneliness and gambling problems is particularly relevant in the context of the proliferation of online-gambling communities, as a recent study in Finland found that those who participate in online gambling communities reported higher loneliness and perceived lack of social connectedness (Sirola et al., 2019).

All together, these results suggest that, although social motives are thought to be less risky than other motives, there is reason to speculate that the relationship between social motives and problem gambling varies in the context of relatedness frustration and feelings of loneliness. There exists a need for examinations of the link between social motives and problem gambling in the context of social deficits, as the possibility exists that the link is contingent on social mechanisms that are rarely the focus on works reporting social motives as non-problematic.

## Present Study

Social motives for gambling have historically been regarded as benign or even protective against gambling problems (Dowling et al., 2021; Francis et al., 2015; Stewart & Zack, 2008) and there is a lack of research focused specifically on social motives and problem gambling. Consequently, few studies have attempted to isolate or examine the specific circumstances under which social motives might be more likely to lead to the development of gambling problems (Elton-Marshall et al., 2018). As such, the present study aims to address gaps in the research regarding the circumstances under which social motives may increase risk of problem gambling, namely deficits in social connectedness or loneliness.

The present work sought to examine whether the link between social motives and problem gambling is contingent on social deficits thought to facilitate problem gambling behavior. As such, the present study aimed to test the interaction between social motives for gambling and loneliness in predicting problem gambling severity. Additionally, the present study also examined whether relatedness frustration moderates the effect of social gambling motives on problem gambling. The present study had two primary aims: (1) test whether social gambling motives and loneliness interact to predict problem gambling severity when controlling for other gambling motives and gambling frequency, and (2) examine whether the relationship between social gambling motives and problem gambling severity varies based on frustration of the need for relatedness (i.e., relatedness frustration) when adjusting for other gambling motives and gambling frequency.

First, it was hypothesized that the interaction of social gambling motives and loneliness would transmit a significant positive effect on problem gambling severity (H1). That is, it was hypothesized that the effect of social gambling motives on problem gambling severity would be strongest at higher (relative to lower) levels of loneliness. Second, we hypothesized that the interaction of social gambling motives and relatedness frustration would transmit a significant positive effect on problem gambling severity. That is, it was hypothesized that the positive predictive effect of social gambling motives on problem gambling severity would be strongest at higher (relative to lower) levels of relatedness frustration (H2).

## Method

### Participants and Procedure

Participants were recruited by YouGov Opinion Polling as a part of a larger study (see Grubbs & Kraus, 2022). In March 2022, a sample of American adults matched to U.S. norms for age, gender, education, census region, and race/ethnicity as of the 2019 American Community Survey (ACS; US Census Bureau, n.d.) (*n* = 2,806, *M*_*age*_*[SD] =* 48.9 [17.2]; 1365[48.6%] men; response rate = 87.6%) and an oversample of sports-wagering adults in the U.S (*n* = 1,557, *M*_*age*_[*SD*] = 41.7[15.3]; 1043[67%] men; response rate = 78.7%) was recruited via YouGov America.

YouGov uses a sample-matching method to construct census-matched samples from a large online opt-in panel. For the present data, YouGov drew a random hypothetical sample based on the full American Community Survey which corresponds to the sampling frame and selects panelists who match the characteristics of the sampling frame member. Specific measures of quality control and responsiveness were guaranteed as a part of YouGov’s proprietary data collection standards. Although YouGov can overestimate rates of problem gambling, and thus should not be used to make epidemiological inferences, it has been found to be useful for understanding gambling behavior at the population level (Sturgis & Kuha, 2021; Wardle & McManus, 2021).

In the present data, the survey routing ensured that gambling measures were completed only by those who endorsed past year gambling of any form (*N* = 2,835). However, the Basic Psychological Need Satisfaction and Frustration Scale (Chen et al., 2015) was administered 1-week post baseline (i.e., Wave 1) of data collection. As such, the moderation analysis for relatedness frustration included only those participants retained 1-week post baseline (*N* = 2051). Further, to be included in the present study, participants were required to provide complete data on demographics (e.g., age, race, income, gender, employment, education). The demographics for the present study’s full sample are available in Table 1.

**Table 1:** Demographics

Variable	*N*	%
Gender		
Female	1155	40.7%
Male	1608	56.7%
Non-binary	42	1.5%
Other	30	1.1%
Age	49.19 (*SD* = 15.70)	
Ethnicity		
White	1918	67.7%
Black	331	11.7%
Hispanic	305	10.8%
Asian	85	3.0%
Native American	61	2.2%
Two or more races	61	2.2%
Other	54	1.9%
Middle Eastern	20	0.7%
Marital Status		
Married	1521	53.7%
Separated	59	2.1%
Divorced	302	10.7%
Widowed	116	4.1%
Never married	667	23.5%
Domestic/civil partnership	170	6.0%
Education		
No HS	71	2.5%
High school graduate	695	24.5%
Some college	540	19.0%
2-year	315	11.1%
4-year	755	26.6%
Post-graduate	459	16.2%
Employment Status		
Full-time	1467	51.7%
Part-time	306	10.8%
Temporarily laid off	18	0.6%
Unemployed	129	4.6%
Retired	532	18.8%
Permanently disabled	174	6.1%
Homemaker	108	3.8%
Student	70	2.5%
Other	31	1.1%
Median Family Annual Income	$60,000-$69,999	

## Measures

### Problem Gambling and Frequency of Gambling

We measured problem gambling using the Problem Gambling Severity Index (PGSI; Ferris & Wynne, 2001). The PGSI includes nine-items that measure symptoms of problem gambling or gambling disorder (e.g., “Have you bet more than you could really afford to lose?;” “Have you felt guilty about the way you gamble or what happens when you gamble?”) on a scale from 0 (*never*) to 3 (*almost always*). A single item was used to measure participants’ frequency of gambling at all, across gambling types, in the last 12 months. Participants reported their frequency of gambling in the last 12 months on a scale from 1 (*never*) to 6 (*more than once per day*). The mean frequency of gambling for the present sample was 3.05 (*SD* = 1.17), corresponding most closely to a frequency of monthly gambling.

### Gambling Motives

We measured motivations for gambling via the Gambling Motives Questionnaire-Financial (GMQ-F; Dechant, 2014). The GMQ-F includes sixteen-items in which participants rate the frequency of different gambling motives using a quasi-interval four-point scale (1 = *never or almost never*; 2 = *sometimes*; 3 = *often*; 4 = *almost always or always*). The GMQ-F includes four subscales: Social, Coping, Enhancement, and Financial Motives. The Social Motives subscale includes 4-items that measure social reasons for gambling (e.g., “To be social”). A full list of the items for each measure used in the present study is available at: https://osf.io/epnxf/?view_only=a219fa5a004b4020b18028024dfa1840.

### Loneliness

We used the UCLA 3-item Loneliness Scale (UCLA-3; Hughes et al., 2004) to measure loneliness. The UCLA-3 is composed of 3 items designed to measure dimensions of loneliness: relational connectedness, social connectedness, and self-perceived isolation. Participants respond to each item (e.g., “How often do you feel that you lack companionship”) using a 3-point scale (1 = *hardly ever*; 2 = *sometime of the time*; 3 = *often*), with higher scores indicating higher loneliness levels.

### Basic Psychological Need Relatedness Frustration

To measure frustration of the basic psychological need for relatedness, the Relatedness Frustration subscale of the Basic Psychological Need Satisfaction and Frustration Scale (Chen et al., 2015) was used in the present study. The BPNSFS is a 24-item questionnaire that was designed to assess individuals’ satisfaction or frustration of three basic psychological needs: Autonomy (i.e., sense of volition and psychological freedom), Competence (i.e., sense of effectiveness and mastery), and Relatedness (i.e., sense of intimacy and connection with others). The BPNSFS includes a satisfaction and frustration subscale for each of the basic psychological needs, culminating in a total of 6 subscales. Given the focus of the present study is on the context of social deficits, only the 4-item Relatedness Frustration subscale (i.e., BPN-RF) was used in the present study. Participants responded to the items (e.g., “I feel the relationships I have are just superficial” and “I feel excluded from the group I want to belong to”) on a scale ranging from 1 (*not true at all*) to 5 (*completely true*).

### Analytic Plan

Preliminary analyses were conducted in SPSS version 28. Prior to testing the proposed moderation models, the focal predictor (social gambling motives) and moderators (loneliness and relatedness frustration) were centered. Descriptive statistics for key variables are available in Table 2. Prior to conducting the primary analyses, measures of internal consistency and bivariate correlations are reported across all measures (See Table 3). The effect sizes found were interpreted using recent benchmarks (e.g., correlations: < 0.10 are very small; < 0.20 are small; <. 30 are moderate, < 0.40 are large, and > 0.40 are very large; Funder & Ozer, 2019).

Two OLS regression moderation analyses were conducted to test whether the relationship between social motives and problem gambling varies based on social deficits, operationalized through self-report measures of loneliness (UCLA-3) and relatedness frustration (BPN-RF). In the first model, loneliness (i.e., UCLA-3) was tested as a moderator in the relationship between social gambling motives and problem gambling. In the second model, relatedness frustration (i.e., BPN-RF) was tested as a moderator in the relationship between social gambling motives and problem gambling. The InterActive application (McCabe et al., 2018) was used to conduct moderation analyses and to plot the interaction results.

**Table 2:** Descriptive statistics for key variables

Variable	Range	M (SD)	Skew	Kurtosis	SE
Social Gambling Motives	1–4	2.054 (0.744)	0.482	− 0.485	0.014
Problem Gambling Severity	0–3	0.439 (0.685)	1.709	1.974	0.013
Loneliness	1–3	1.646 (0.631)	0.634	− 0.684	0.012
Relatedness Frustration	1–20	8.588 (4.170)	0.659	− 0.494	0.092

## Results

Consistent with expectations, results demonstrated a positive large correlation between social gambling motives and problem gambling severity. Loneliness was found to have a positive moderate correlation with problem gambling, while relatedness frustration was found to have a positive large correlation with problem gambling. The correlation between social motives and loneliness was positive and small in magnitude, while the correlation between social motives and relatedness frustration was positive and moderate in magnitude. A large, positive correlation between loneliness and relatedness frustration was also found. Of the gambling motives, coping was found to have the strongest correlation with problem gambling, followed by social motives, financial motives, then enhancement motives. Gambling frequency was found to be positively correlated with all the study variables.

**Table 3:** Pearson’s correlations and internal consistencies for all Key measures

Variable	1	2	3	4	5	6	7	8
(1) (1) Social Motives	1							
(2) Problem Gambling Severity	0.460^**^	1						
(3) Loneliness	0.131^**^	0.314^**^	1					
(4) Relatedness Frustration	0.302^**^	0.475^**^	0.516^**^	1				
(5) Coping Motives	0.604^**^	0.693^**^	0.249^**^	0.433^**^	1			
(6) Financial Motives	0.327^**^	0.359^**^	0.149^**^	0.236^**^	0.492^**^	1		
(7) Enhancement Motives	0.542^**^	0.327^**^	0.071^**^	0.157^**^	0.538^**^	0.473^**^	1	
(8) Gambling Frequency	0.197^**^	0.350^**^	0.057^**^	0.160^**^	0.331^**^	0.284^**^	0.340^**^	1
Mean	2.05	0.44	1.64	8.59	1.73	2.38	2.47	3.05
SD	0.74	0.69	0.63	4.17	0.79	0.81	0.78	1.17
Cronbach’s α	0.78	0.95	0.84	0.84	0.86	0.77	0.82	NA
McDonald’s ω	0.79	0.95	NA	0.84	0.86	0.76	0.82	NA

^*^Correlation is significant at the 0.05 level (2−tailed)

^**^Correlation is significant at the 0.01 level (2−tailed)

### Moderation Analyses Results

**Fig. 1:**
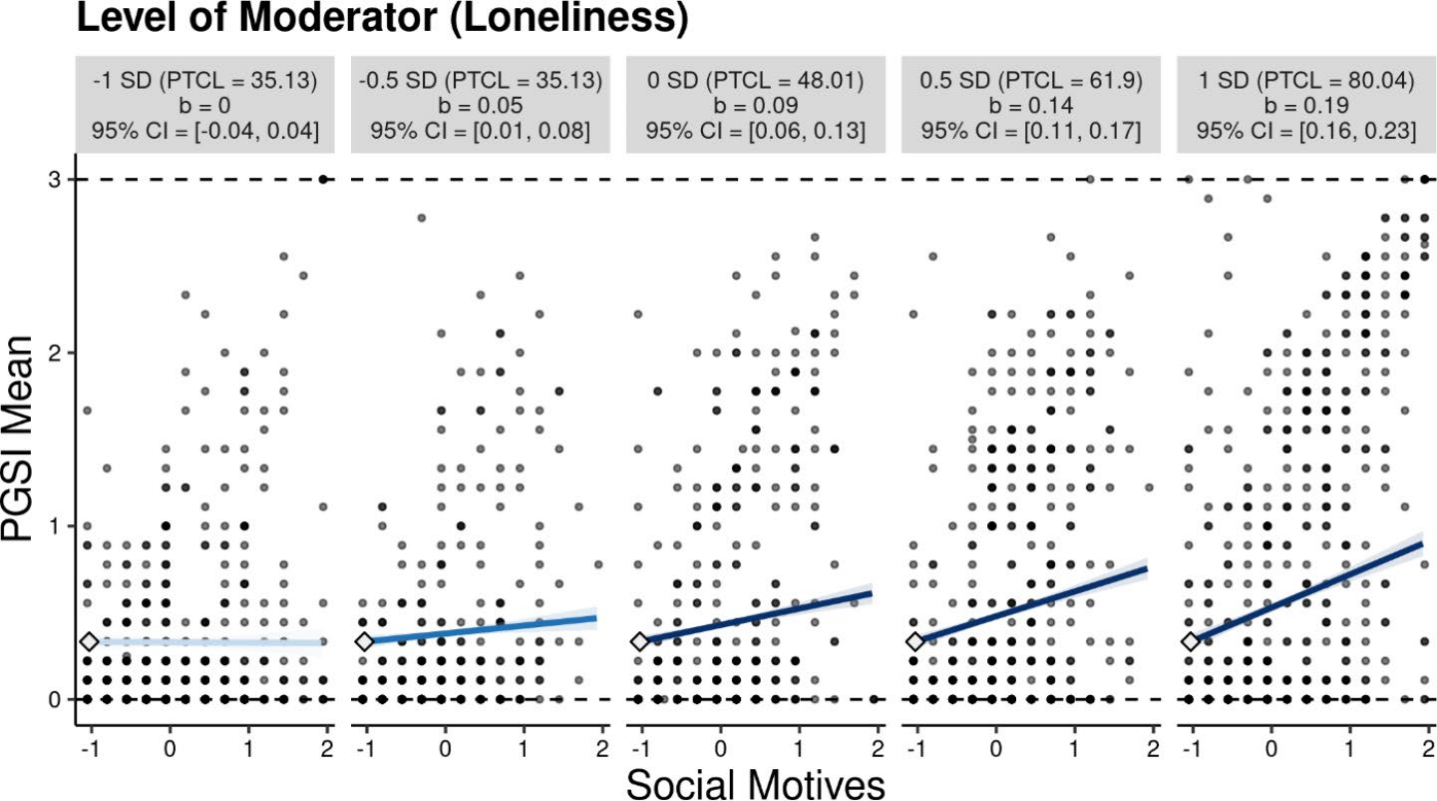
Visualization of Moderation Analysis for Loneliness

**Fig. 2:**
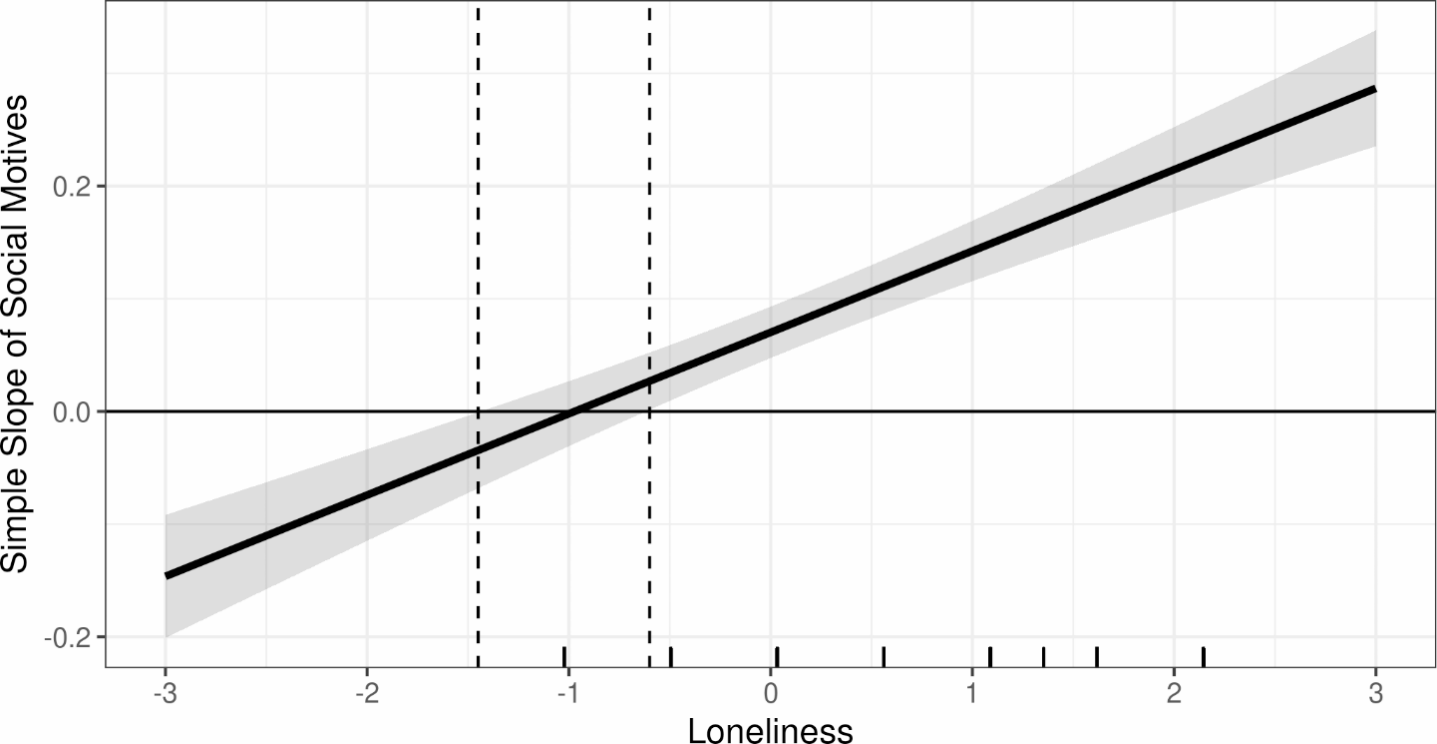
Marginal Effects Plot for Loneliness Interaction

The present study included a test of both loneliness (i.e., UCLA-3) and relatedness frustration (i.e., BPN-RT) as separate moderators in the effect of social gambling motives and problem gambling severity while adjusting for other motives for gambling and gambling frequency (see Tables 4 and 5 for OLS regression moderation results). In the model in which loneliness was the moderator, results demonstrated social motives had a small positive effect on problem gambling (*b* = 0.094, *p* = < 0.001, 95%, CI = [0.064, 0.125], *β* = 0.103) when adjusting for the other motives and gambling frequency. In addition, loneliness was found to transmit a positive effect on problem gambling (*b* = 0.158, *p* = < 0.001, 95%, CI = [0.130, 0.187], *β* = 0.146). Consistent with H1, findings demonstrated that the interaction of social gambling motives and loneliness transmitted a significant positive effect on problem gambling (*b* = 0.154, *p* = < 0.001, 95%, CI = [0.120, 0.188], *β* = 0.115). The moderation accounted for 1.3% of the variance in problem gambling (*R*^*2*^ = 0.013, *F* = 79.007, *p* < .001) and was found to be conditioned on loneliness scores. A visualization of the significant interaction (-1 *SD* to + 1 *SD*) is available in Fig. 1. Figure 1 demonstrates that for participants low in loneliness (i.e., -1 *SD*), social gambling motives are not significantly associated with an increase in PGSI scores. However, social motives are significantly positively associated with an increase in PGSI scores at mean levels of loneliness (i.e., 0 SD). Further, for participants high in loneliness (i.e., + 1 *SD*), a one unit increase in social gambling motives was associated with a 0.19 increase in PGSI mean scores. Specifically, findings demonstrated that a positive effect of social motives on problem gambling is conditioned on levels of loneliness. Figure 2 shows a marginal effect plot indicating that the effect of social motives on PGSI mean scores is only positive and significant at -0.6 *SD* or greater (i.e., more positive) loneliness values.

**Fig. 3:**
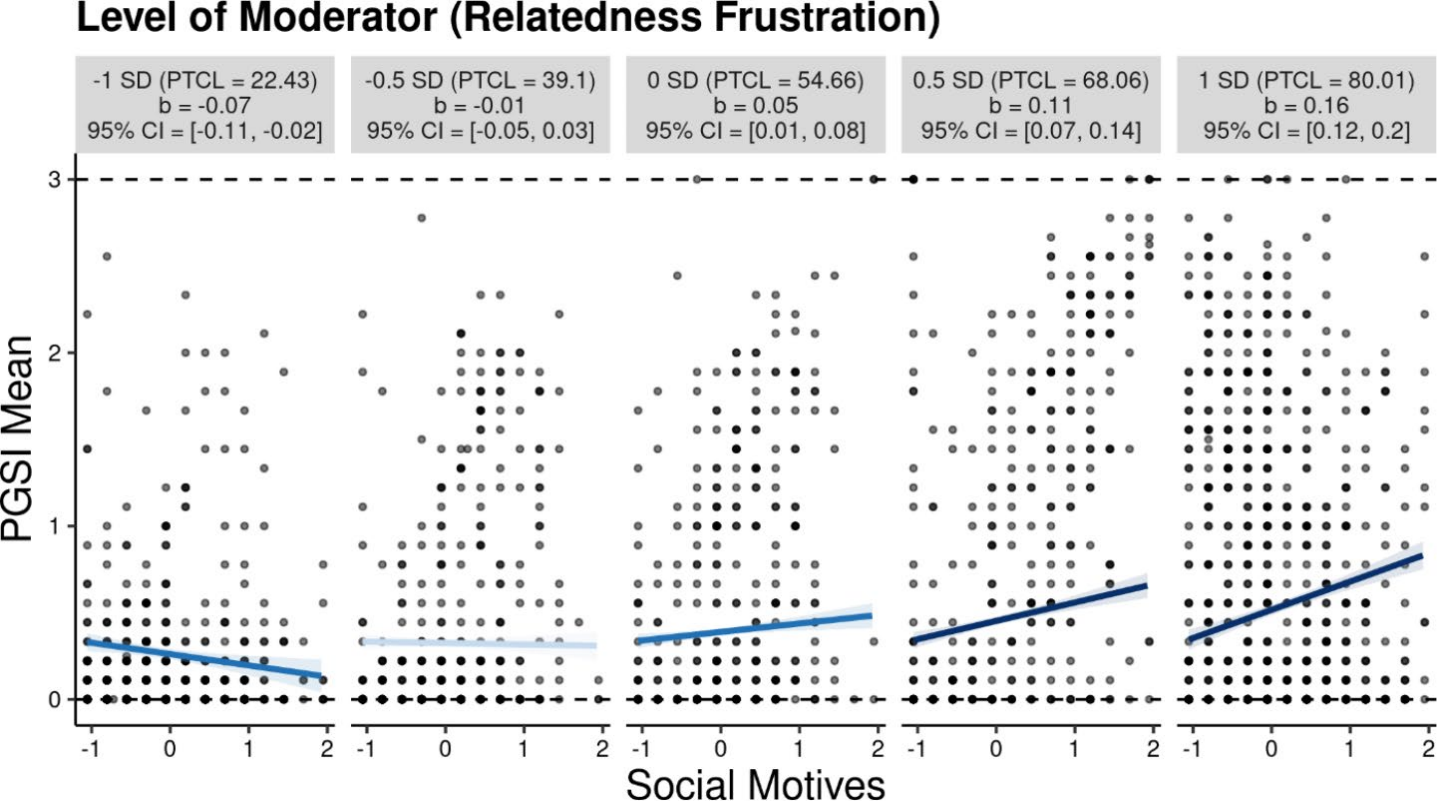
Visualization of Moderation Analysis for Relatedness Frustration

**Fig. 4:**
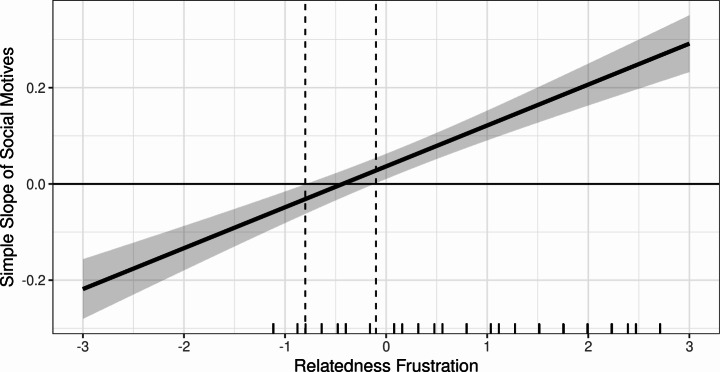
Marginal Effects Plot for Relatedness Frustration Interaction

With regards to relatedness frustration as the moderator, findings showed that social motives had a small positive effect on problem gambling (*b* = 0.049, *p* = < 0.05, 95%, CI = [0.014, 0.084], *β* = 0.054) when adjusting for the other motives and gambling frequency. Further, relatedness frustration was found to transmit a positive effect on problem gambling (*b* = 0.122, *p* = < 0.001, 95%, CI = [0.101, 0.143], *β* = 0.194). Consistent with H2, findings demonstrated that the interaction of social gambling motives and relatedness frustration transmitted a significant positive effect on problem gambling (*b* = 0.109, *p* = < 0.001, 95%, CI = [0.086, 0.133], *β* = 0.144). The moderation accounted for 1.9% of the variance in problem gambling (*R*^*2*^ = 0.019, *F* = 83.551, *p* < .001) and was found to be conditioned on relatedness frustration scores. A visualization of the significant interaction (-1 *SD* to + 1 *SD*) is available in Fig. 3. Figure 3 demonstrates that for participants low in relatedness frustration (i.e., -1 *SD*), social gambling motives are significantly negatively associated with a decrease in PGSI scores (*b* = -0.07, *p* = 95%, CI = [-0.11, -0.02]. However, at relatedness frustration levels − 0.5 *SD* below the mean, the effect is no longer significant. Further, for participants high in relatedness frustration (i.e., + 1 *SD*), a one unit increase in social gambling motives was associated with a 0.16 increase in PGSI mean scores. Specifically, findings demonstrated that a positive effect of social motives on problem gambling is conditioned on relatedness frustration levels. Figure 4 shows a marginal effect plot indicating that the effect of social motives on PGSI mean scores is only significant and negative when relatedness frustration is -0.80 *SD* away from the mean or further and is only significantly positive when relatedness frustration is 0 *SD* or greater (i.e., more positive).

**Table 4:** OLS results with loneliness (UCLA-3) as the moderator

Variable	b	se	β	t	*p*	LLCI	ULCI
PGSI: *R* = .738, *R*^*2*^ = 0.544, *MSE =* 0.215, *F*(7, 2827) = 482.660, *p* < .001							
GMQ-S	0.094	0.016	0.103	6.058	< 0.001	0.064	0.125
Loneliness	0.158	0.014	0.146	11.080	< 0.001	0.130	0.187
GMQ-S x UCLA-3	0.154	0.017	0.115	8.889	< 0.001	0.120	0.188
GMQ-C	0.505	0.016	0.585	31.950	< 0.001	0.474	0.536
GMQ-F	0.022	0.013	0.026	1.668	0.095	− 0.004	0.047
GMQ-E	− 0.109	0.015	− 0.124	-7.286	< 0.001	− 0.138	− 0.080
Gambling Frequency	0.087	0.008	0.149	10.699	< 0.001	0.071	0.103

**Table 5:** OLS results with relatedness frustration (BPN-RF) as the moderator

Variable	b	se	β	t	*p*	LLCI	ULCI
PGSI: *R* = .734, *R*^*2*^ = 0.539, *MSE =* 0.202, *F*(7, 2043) = 341.224, *p* < .001							
GMQ-S	0.049	0.018	0.054	2.720	0.007	0.014	0.084
BPN-RF	0.122	0.011	0.194	11.370	< 0.001	0.101	0.143
GMQ-S x BPN-RF	0.109	0.012	0.144	9.141	< 0.001	0.086	0.133
GMQ-C	0.440	0.019	0.520	23.288	< 0.001	0.403	0.477
GMQ-F	0.010	0.015	0.012	0.657	0.511	− 0.019	0.039
GMQ-E	− 0.087	0.017	− 0.102	-5.113	< 0.001	− 0.121	− 0.054
Gambling Frequency	0.082	0.010	0.138	8.326	< 0.001	0.063	0.101

## Discussion

The present study aimed to examine whether the strength of the effect of social motives on problem gambling varies based on deficits in social context, specifically loneliness and relatedness frustration. Findings supported our first hypothesis, with results demonstrating that the interaction of social gambling motives and loneliness transmitted a significant positive effect of problem gambling severity. Further, findings provided insight into the conditions under which social motives have a positive effect on problem gambling, with findings showing that social motives only had a positive effect on problem gambling severity at higher levels of loneliness. That is, findings indicated that, for individuals who do not experience significant levels of loneliness, social motives may not be problematic; however, among individuals with higher loneliness levels (-0.6 *SD* or greater), social motives are predictive of greater problem gambling severity.

Findings also supported hypothesis 2, with results showing that the interaction of social gambling motives and relatedness frustration transmitted a significant positive effect of problem gambling severity. In addition, findings demonstrated that the effect of social motives on problem gambling is contingent on levels of relatedness frustration. Specifically, findings indicated that, at lowest levels of relatedness frustration (-1 *SD*), social motives are negatively associated with problem gambling severity but, at moderately low levels of relatedness frustration, social motives do not transmit a significant effect on problem gambling. On the contrary, findings showed that the effect of social motives on problem gambling is only positive at mean (0 *SD*) or greater levels of relatedness frustration, with the strength of the effect increasing at higher relatedness frustration levels.

### The Role of Social Deficits in Problem Gambling

Findings provided unique insights regarding the role of loneliness in amplifying the effect of social gambling motives on problem gambling. Specifically, findings suggest that gambling motivated by social factors may be associated with greater risk of problems in the context of loneliness or social isolation. Likewise, findings indicate that those who gamble for social reasons but do not experience such feelings of loneliness are less susceptible to problem gambling. Findings suggest that those who feel lonely may gamble more to escape feelings of loneliness or to seek social connection, which can lead to greater problem gambling behavior. This is consistent with previous research indicating that loneliness predicts problem gambling and emphasizing the importance of considering individual’s social needs as factors maintaining problem gambling (Botterill et al., 2016; Elton-Marshall et al., 2018; McQuade & Gill, 2012).

Consistent with previous works demonstrating that frustration of basic psychological needs is associated with problem gambling (Vuorinen et al., 2022), whereas basic psychological needs satisfaction is associated with greater adherence to responsible gambling practices and less risk of gambling problems (Dennis et al., 2017; Tong et al., 2022), the present study’s findings support that basic psychological needs influence the trajectory of gambling-related problems. Findings also supported that the strength and direction of the effect of social motives on problem gambling severity was conditioned on frustration levels of the basic psychological need for relatedness. Specifically, results suggest that, for those that do not report significant relatedness frustration, social motives were associated with lower risk of problem gambling. However, results suggested that social motives are predictive of greater risk of problem gambling among those who report higher levels of relatedness frustration.

### Social Motives in Context

The present work expanded on prior gambling research by examining whether social context could provide insights into the circumstances in which social motives are associated with problem gambling. Collectively, results underscore the role of social deficits (i.e., loneliness and relatedness frustration) as motivating factors that can increase risk of gambling problems (Vuorinen et al., 2022) and highlight the importance of studying gambling motives and risk of problems in the context of deficits using an SDT framework (Hagfors et al., 2023). In the case of loneliness, findings indicate that whether social motives predict problems may depend on subjective feelings of being alone or lacking social connection. Regarding relatedness frustration, findings suggest that whether social motives are a protective factor for problem gambling (Mackinnon et al., 2017) or associated with greater risk of problem gambling (Estévez et al., 2021; Gökce Yüce et al., 2022) may be contingent on the degree to which one feels unfulfilled or dissatisfied in their relationships with others.

### Implications

The present study has theoretical implications regarding the notion that social motives as normative and a healthier, less risky reason for gambling (Barrada et al., 2019; Mackinnon et al., 2017), as findings demonstrated possible contexts or circumstances in which social motives appear more likely to lead to the development of problem gambling. That is, findings expand on prior motivational theories of problem gambling by providing evidence that, in the context of social deficits (i.e., loneliness and relatedness frustration), social motives predict problem gambling when adjusting for other motives and gambling frequency. While it is unlikely that social motives are the most robust predictor of problem or disordered gambling across contexts, in the context of social deficits, social motives may contribute to the initiation or maintenance of gambling-related problems. As recommended by Vuorinen et al. (2022), the present study also addresses the need to separately examine the individual role of each basic psychological need, as opposed to using a composite measure aggregating each of the BPNSFS subscales, in problem gambling. Lastly, the present work adds to a growing body of research indicating the utility of examining problem gambling using an SDT framework, with results supporting that gambling-related problems can, in part, result from frustration of basic psychological needs.

Findings also have clinical implications regarding problem gambling treatment and prevention strategies. Specifically, findings underscore the importance of tailoring gambling treatment and prevention strategies among those whose problematic gambling behavior is maintained by social motives stemming from social deficits or unmet social needs (Botterill et al., 2016; McQuade & Gill, 2012). In such cases, it may be beneficial to make addressing feelings of loneliness or social needs a treatment focus to promote sustainable treatment effects, such as helping individuals identify alternative, more adaptive ways to connect with others and find need-supportive environments. Gambling treatment programs that incorporate social support interventions or peer-based community engagement could help alleviate the social deficits that maintain excessive gambling.

While previous research has found motivation-matched approaches to treatment to be effective in treating problem gambling (Stewart et al., 2016), to our knowledge, no such treatment protocols exist which consider social motives, likely given notions that social motives are largely non-problematic. The present work points to the need for not only more research examining social motives and problem gambling within social context, but also the need for the development of treatment and intervention strategies tailored to those whose problem gambling is maintained by unfulfilled social needs. Given the support found for the moderating influence of social deficits in the relationship between social motives and problem gambling, future research should explore how to integrate strategies targeting these social factors into existing treatment frameworks.

### Limitations

The present study has noteworthy limitations. First, the data used was cross-sectional and therefore is not sufficient for making causal inferences. As underscored in recent works (Hagfors et al., 2023), there exists a need for longitudinal works regarding the relationship between motives, deficits in basic psychological needs, and problem gambling to examine the relationships overtime and to examine the relative stability of motives overtime (Hagfors et al., 2023). Further, there are established limitations associated with the present study’s use of self-report measures (Chan, 2009). Lastly, the present study’s sample endorsed higher rates of problem gambling than reported in other population estimates and thus should not be used to make epidemiological claims.

## Conclusion

The present study examined the moderating influence of social deficits (i.e., loneliness and relatedness frustration) on the relationship between social motives for gambling and problem gambling. In doing so, it addressed key gaps in the literature, including the lack of research on social motives—historically regarded as benign or protective—and the need to identify circumstances in which social motives are linked to greater risk of problem gambling, challenging assumptions about their minimal relevance to gambling-related harm. Consistent with expectations, findings demonstrated that both the interaction of social motives and loneliness and the interaction of social motives and relatedness frustration transmitted a significant effect on problem gambling. In addition, findings demonstrated that a significant positive effect of social motives on problem gambling was conditioned on higher levels of loneliness. Findings also showed that the strength and direction of the effect of social motives on problem gambling severity was conditioned on relatedness frustration levels, with findings indicating that social motives may be a protective factor for problem gambling at low relatedness frustration but are a risk factor for problem gambling at higher levels of relatedness frustration. The results of the present study suggest that the link between social motives for gambling and problem gambling may be contingent on social deficits, namely feelings of loneliness and having unmet social needs.

## Data Availability

The data and supplementary materials are available from the open science framework database (link: https://osf.io/epnxf/?view_only=a219fa5a004b4020b18028024dfa1840).
